# Current Immunotherapy Strategies for Rheumatoid Arthritis: The Immunoengineering and Delivery Systems

**DOI:** 10.34133/research.0220

**Published:** 2023-10-17

**Authors:** Chenyu Zhang, Peixiang Ma, An Qin, Liao Wang, Kerong Dai, Yuanyuan Liu, Jie Zhao, Zuyan Lu

**Affiliations:** ^1^ School of Medicine, Shanghai University, Shanghai, China.; ^2^ Clinical and Translational Research Center for 3D Printing Technology, Shanghai Ninth People's Hospital, Shanghai Jiao Tong University School of Medicine, Shanghai, China.; ^3^ Shanghai Key Laboratory of Orthopedic Implants, Department of Orthopedic Surgery, Shanghai Ninth People's Hospital, Shanghai Jiao Tong University School of Medicine, Shanghai, China.; ^4^ Shanghai Frontiers Science Center of Degeneration and Regeneration in Skeletal System, Shanghai, China.

## Abstract

Rheumatoid arthritis (RA) is a chronic inflammatory autoimmune disease accompanied by persistent multiarticular synovitis and cartilage degradation. The present clinical treatments are limited to disease-modifying anti-rheumatic drugs (DMARDs) and aims to relieve pain and control the inflammation of RA. Despite considerable advances in the research of RA, the employment of current clinical procedure is enormous, hindered by systemic side effect, frequent administration, tolerance from long-lasting administration, and high costs. Emerging immunoengineering-based strategies, such as multiple immune-active nanotechnologies via mechanism-based immunology approaches, have been developed to improve specific targeting and to reduce adverse reactions for RA treatments. Here, we review recent studies in immunoengineering for the treatment of RA. The prospect of future immunoengineering treatment for RA has also been discussed.

## Introduction

Rheumatoid arthritis (RA) as an autoimmune disease, characterized by synovial inflammation and hyperplasia, affects 0.5 to 3% of the world’s population and primarily manifests as chronic inflammatory arthropathy [1]. Despite advances in treatment, patients still experience significant pain, joint damage, and disability [2]. The RA patient also requires ongoing medical care, medications, and therapies; these socioeconomic costs manifest with rising health care costs and the stretching of resources [3]. Despite significant improvements in RA therapy, the existing therapeutic techniques are greatly hampered by various flaws, such as significant systemic adverse effects, frequent and chronic administration routines, and the tolerance built by them [4]. Immunoengineering therapy is a strategy based on the molecular pathological microenvironment of RA to enhance therapeutic effects, which may bring a breakthrough for the future of immunotherapy to treat RA [5]. In this review, we seek to summarize the key molecular mechanisms for RA pathology processes and the present immunoengineering strategies, which would pave the way for designing more effective therapies for RA in the future.

## Biological Pathways for RA Pathological Processes

**Fig. 1:**
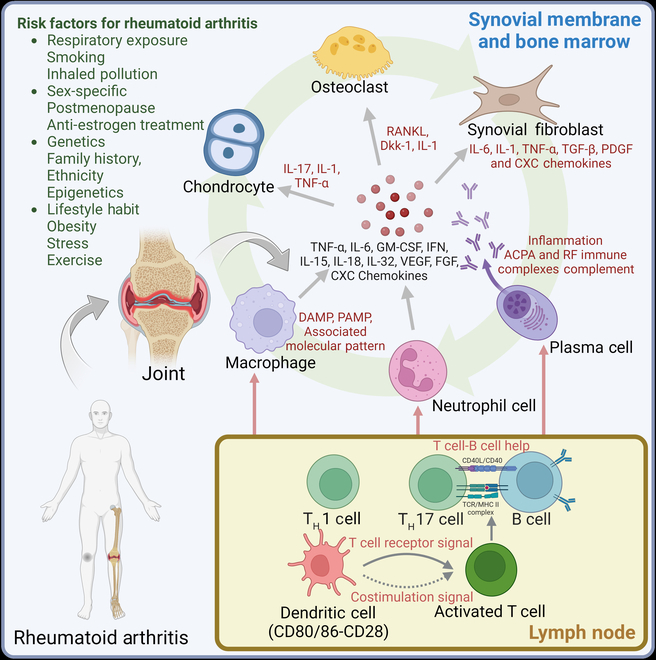
Biological pathways and risk factors for RA pathological processes.

Although several risk factors (such as genes, gender, age, obesity, and infections) have been identified recently (Fig. 1), the exact cause of RA is still unknown [6]. It has been reported that TA could be triggered by the interaction between the predisposing genes (HLA-DRβ1) [7] and the environmental factors (cigarette smoke) [8], which could promote the onset of citrullination (deimination) of the intracellular protein [9] and matrix protein, resulting in the production of anti-citrullinated protein antibodies (ACPAs), rheumatoid factor (RF), and the initiating of the inflammatory and destructive synovial response [10]. Then, the immune cells infiltrated into the normal synovial membrane, interacting to produce an inflammatory cascade, which is characterized by the interactions of fibroblast-like synoviocytes (FLSs) with the cells of the innate immune system, including monocytes, dendritic cells (DCs), T lymphocytes, and B cells. The immune cells would secrete the proinflammatory cytokines, such as interleukin (IL-1, IL-6, and IL-17), tumor necrosis factor–α (TNF-α), matrix metalloproteinases (MMPs), and granulocyte–macrophage colony-stimulating factor (GM-CSF), which further facilitate the infiltration of monocytes into the joint [11]. Specifically, the T cells could activate antigen-presenting cells (APCs) secretions through CD80/CD86 that interact with CD28 of T cells, subsequently secreting IL-17, IL-1β, and IL-6, and activating the B cell through CD40L and the macrophages to exacerbate the synovial inflammation in synovium [12]. The macrophages also stimulate the osteoclasts to induce bone resorption and cartilage degradation by secreting IL-6 and GM-CSF. Besides, the imbalance between proinflammatory M1 macrophage and anti-inflammatory M2 macrophage has also contributed to the inflammatory processes in RA [13]. Eventually, under the fulminant stage, the synovium undergoes synovial hyperplasia, joint swelling, and angiogenesis, which eventually result in the degeneration of the articular cartilage in RA [14].

Overall, the cellular pathways involved in RA are complex and interconnected, involving multiple cell types and signaling pathways. Understanding these pathways is crucial for the study of the pathological development of RA, such as the specific cytokines or immune cells involved in the disease process. Moreover, targeting these cytokines with biological therapies might lead to significant improvements in disease treatment for many patients.

## Current Immunotherapies for RA

The clinical treatment for RA is divided into conventional synthetic disease-modifying anti-rheumatic drugs (cDMARDs) [15], biological DMARDs (bDMARDs), nonsteroidal anti-inflammatory drugs (NSAIDs), corticosteroids, and cell-based therapy.

### cDMARDs

cDMARDs are traditional medications to treat RA, which could modify the course of the disease and slow down the progression of joint damage and inflammation caused by the overactive immune system. The common cDMARDs used for RA include methotrexate (MTX), sulfasalazine, aurothiomalate (gold salts), leflunomide, and hydroxychloroquine. Up to now, cDMARDs remain an essential component of RA management, especially for those who have milder forms of the disease or cannot tolerate other medications (Table 1).

**Table 1.** Clinical cDMARD therapies for RA and their target and mechanism.

Drug	Structure	Target	Mechanism
Methotrexate	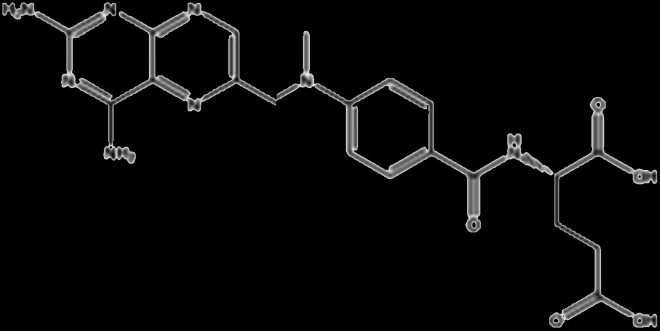	Folate-dependent enzymes (DHFR)	Impaired DHFR leads to inhibition of lymphocyte proliferation [87]
Leflunomide	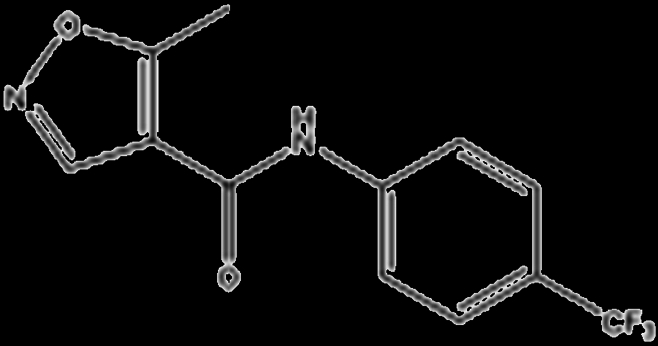	Dehydroorotate dehydrogenase (DHODH)	Inhibits pyrimidine de novo synthesis, leads to inhibition of lymphocyte proliferation [88]
Sulfasalazine	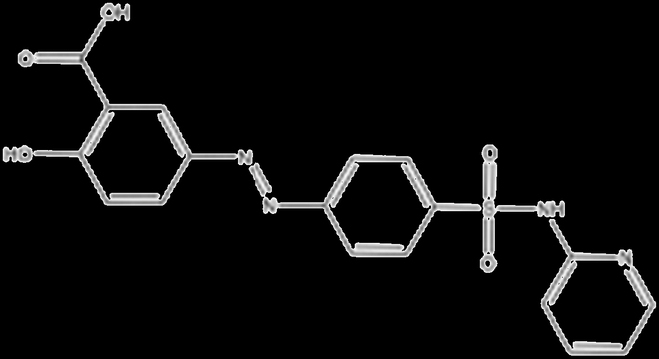	Folate-dependent enzymes (DHFR)	Suppresses folate-dependent pathways in de novo synthesis of DNA [89]
Aurothiomalate	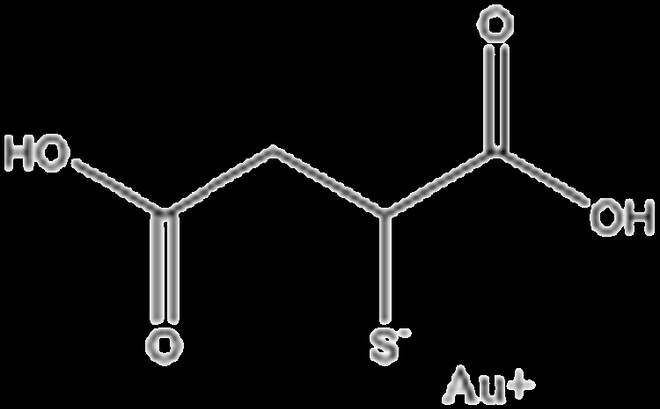	Broad immunosuppression	Inhibition of signal transduction and antigen presentation
Hydroxychloroquine	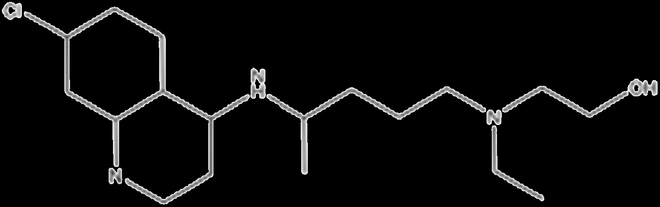	Lysosomes	Hinders antigen presentation and impairs activation of the innate immune system [88]
Tofacitinib	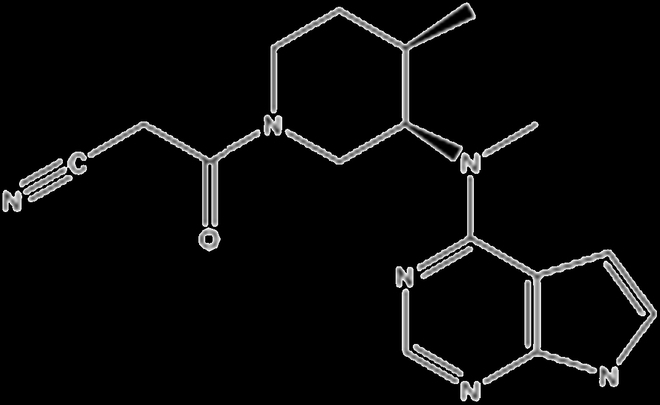	Janus kinase 1 (JAK1) and Janus kinase 3 (JAK3)	Produces small increases in lipid levels, suggesting implications in lipid metabolism [90]

### bDMARDs

bDMARDs, as a new class of DMARDs, are a type of immunosuppressive therapy that specifically targets certain molecules (such as IL-1, TNF-α, and IL-6) involved in the immune response. Unlike cDMARDs, which are typically small-molecule drugs, bDMARDs are large, complex molecules that are often administered through injection or infusion and have a more targeted approach in treating RA by blocking specific immune signaling pathways that contribute to inflammation and joint damage (Table 2).

**Table 2.** Approved bDMARDs to treat RA.

Agent	Class	Target	Structure	Mechanism	Administration
Etanercept	Cytokine inhibitor	TNF-α	TNF-α receptor–Fc fusion	Decoy receptor binding to soluble TNF [91]	sc injection
Infliximab	Cytokine inhibitor	TNF-α	Chimeric murine–human IgG1 monoclonal Ab	Ab binding to TNF [92]	iv infusion
Anakinra	Cytokine inhibitor	IL-1	Recombinant human IL-1 receptor antagonist	Binding to IL-1 type-1 receptor [93]	sc injection
Adalimumab	Cytokine inhibitor	TNF-α	Human monoclonal antibody	Ab binding to TNF [94]	sc injection
Abatacept	Costimulation blocker	CD80 and CD86	CTLA-4 IgG1 fusion protein	T cell costimulation blocker [95]	iv infusion or sc injection
Rituximab	Cell-depleting agent	CD20	Chimeric murine–human monoclonal IgG1k Ab	Binding to and depletion of CD20-positive B cells [96]	iv infusion
Certolizumab pegol	Cytokine inhibitor	TNF-α	Pegylated humanized Fab fragment of an anti-TNF-α monoclonal antibody	Fab fragment binding to TNF [97]	sc injection
Golimumab	Cytokine inhibitor	TNF-α	Human monoclonal antibody	Ab binding to TNF [98]	sc injection
Tocilizumab	Cytokine inhibitor	IL-6	Recombinant humanized anti-human IL-6 receptor monoclonal Ab	Binding to a soluble membrane-bound IL-6 receptor [99]	iv infusion

sc, subcutaneous injection; iv, intravenous injection.

**Table 3.** Summary of present clinical trials for MSC in RA.

Clinical trial no.	Clinical phase	Source	Registration (year)	Completion date	Country	Statue	RA patients	Administration	Doses	Follow-up (month)	Control group
NCT03333681	Phase 1	BM	2018	2017Nov	N/A	Completed	Refractory	Autologous	1/2 ×10^6^ cells/kg	6 m	No-single group assignment
NCT03067870	Phase 1	BM	2017	2017Mar	N/A	Unknown	N/A	Autologous	Unknown	6 m	No-single group assignment
NCT01985464	Phase 1, 2	UC	2013	2013Nov	Panama	Active, not recruiting	Refractory	Allogeneic	Unknown	12 m	No-single group assignment
NCT00278551	Phase 1	HP	2006	2011Nov	United States	Terminated	Refractory	Autologous	1.4 x 10^6^ CD34^+^ cells/kg	60	No-single group assignment
NCT04170426	Phase 1, 2	AD	2020	2023Dec	United States	Active, not recruiting	Refractory	Autologous	2.0-2.86×10^6^ cells/kg on days 1, 4, and 7	12	Yes
NCT01547091	Phase 1, 2	UC	2012	2014Oct	China	Unknown	N/A	Allogeneic	Single dose, 4x10^7^ cells	12	No-single group assignment
NCT01873625	Phase 2, 3	BM	2013	2011Mar	Iran	Completed	N/A	Autologous	N/A	12	Yes
NCT02643823	Phase 1	UC	2015	2017Jan	China	Unknown	N/A	Allogeneic	2×107; repeat every week for four times	12	Yes
NCT01663116	Phase 1, 2	AD	2012	2019Apr	Spain	Completed	Refractory	Allogeneic	1/2x10^6^ cells/kg at days 1, 8, and 15	6	Yes
NCT04971980	Phase 1, 2	UC	2021	2022Dec	China	Recruiting	Refractory	Allogeneic	0.5/1.0/1.5x10^6^ cells/kg body weight	1	No-sequential assignment
NCT03691909	Phase 1, 2	AD	2018	2020 Aug	United States	Recruiting	Stable Treatment	Autologous	N/A	12	No-single group assignment
NCT00282412	Phase 1	HP	2006	2016Jun	United States	Terminated	Refractory	Allogeneic	N/A	6	No-single group assignment
NCT05003934	Phase 1	UC	2021	2025Sep	Argentina	Recruiting	N/A	Allogeneic	100 million cells for single dose	48	No-single group assignment
NCT03186417	Phase 1	N/A	2017	2022Apr	United States	Recruiting	Refractory	Allogeneic	2/4/6 million hMSC/kg	12	No-sequential assignment
NCT03798028	N/A	UC	2019	2019Dec	China	Recruiting	Anemia or pulmonary disease associated	Allogeneic	N/A	1×10^6^ cells/kg	6
NCT02741362	Phase 1	AD	2016	2017Mar	United States	Terminated	Refractory	Autologous	0.75/1.5×10^6^ cells/kg	6	No-single group assignment
NCT03828344	Phase 1	UC	2020	2024Dec	United States	Active, not recruiting	Refractory	Allogeneic	N/A	12	Yes
NCT02348086	N/A	AD	2015	2018Aug	United States	Unknown	N/A	Autologous	N/A	12	NA
NCT01978639	N/A	BM	2013	2017May	United States	Unknown	N/A	Autologous	0.25/1.0x10^7^ stem cells	6	NA
NCT02221258	Phase 1	UC	2014	2015Oct	Korea	Completed	Refractory	Allogeneic	N/A	1	No-single group assignment
NCT00010335	Phase 1	HP	2001	2015Mar	United States	Completed	Refractory	Autologous	N/A	60	No-single group assignment
NCT01885819	Phase 1, 2	AD	2013	2017Aug	Panama	Withdrawn	Refractory	Autologous	0.5/1.0 x 10^8^ cells /body 3 repeated at 4 weeks intervals	6	No-single group assignment
NCT03618784	Phase 1, 2	UC	2018	2022Oct	Korea	Completed	Refractory	Allogeneic	N/A	4	Yes
NCT00006055	N/A	HP	2000	2005Jun	United States	Unknown	Refractory	Autologous	N/A	60	NA
NCT01413061	N/A	AD	2010	2017Feb	United States	Completed	N/A	Autologous	N/A	24	Yes
NCT02759731	Phase 1, 2	HP	2016	2022Jun	United States	Active, not recruiting	N/A	Allogeneic	N/A	6	Yes
NCT04528355	N/A	HP	2020	2023Feb	United States	Recruiting	N/A	N/A	NA	60	N/A

BM, bone marrow; UC, umbilical cord; HP, hematopoietic; AD, adipose.

### Mesenchymal stem cell therapy and immune cell therapy

Despite recent breakthroughs in cDMARDs (represented by chemical small molecules) and bDMARDs (represented by biological macromolecules), a significant proportion (5 to 20%) of people with RA are intolerant or resistant to these treatments [16–18]. Cell treatment [particularly mesenchymal stem cell (MSC) therapy and macrophage therapy] has shown promise in preclinical trials for lowering inflammation and boosting tissue repair.

**Fig. 2.**
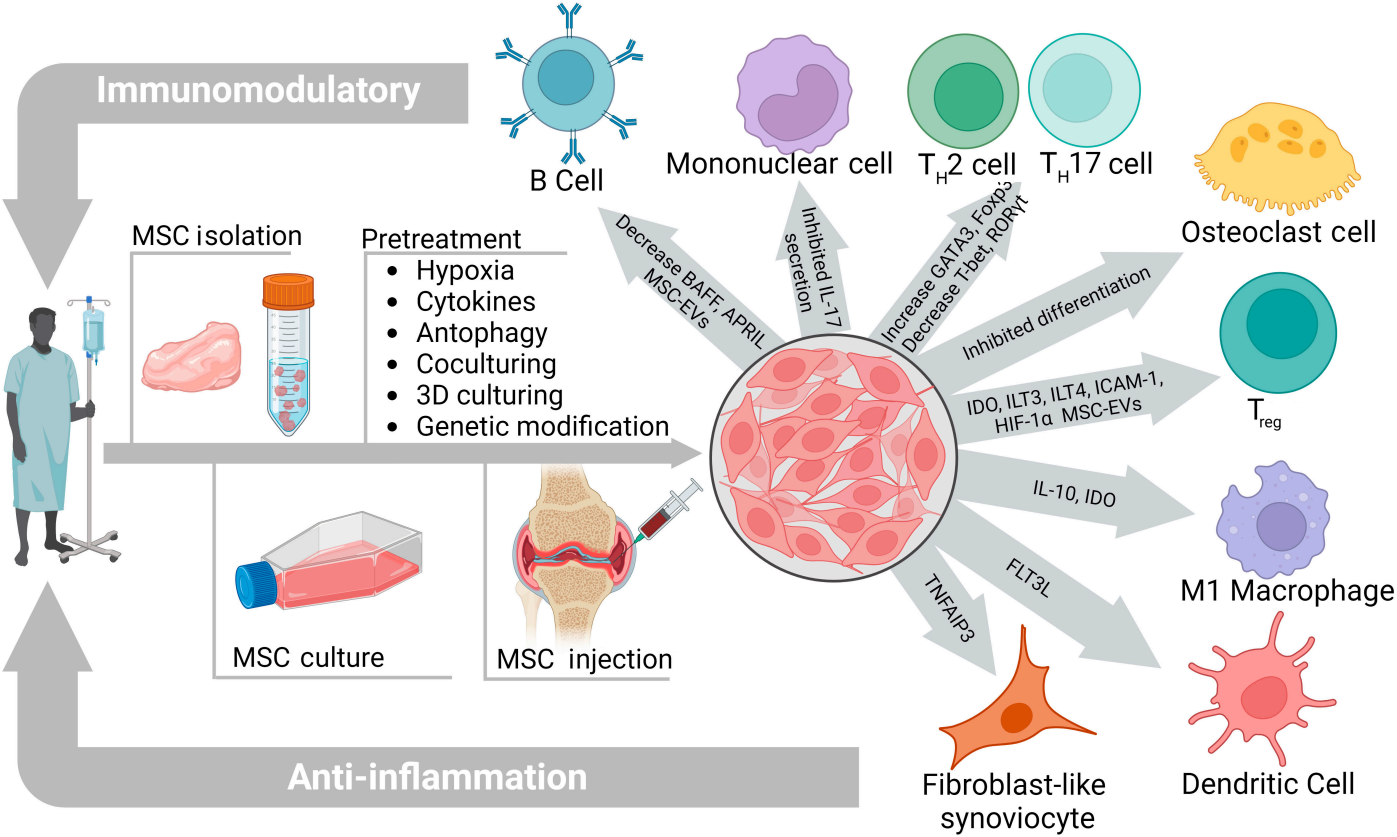
The schematic illustration of immunomodulatory therapy of MSC in treating patients with RA. The mechanism of MSCs treated by different approaches to enhance immunomodulatory and mediate the pathological processes of RA. BAFF, B cell activating factor; APRIL, a proliferation-inducing ligand; EV, extracellular vesicles; IDO, indoleamine 2,3-dioxygenase; ILT, immunoglobulin-like transcript; ICAM-1, intercellular adhesion molecule-1; FLT3L, FMS-like tyrosine kinase-3 ligand; TNFAIP3, tumor necrosis factor–α-induced protein 3.

MSC is a type of stem cell that can differentiate into different cell types. For the immunomodulatory function of MSCs, numerous mechanisms for immunological responses have been reported (Fig. 2). MSCs can mediate powerful immunoregulatory effects by inducing factors, such as prostaglandin E_2_ [19], TGF-β [20], and IL-10 [21]. MSCs have also been reported to induce the macrophages from M1 into M2 phenotype [22]. Furthermore, MSCs have been demonstrated to reduce the expression of APRIL and BAFF to reduce B cell response. It could also up-regulate T helper 2 (T_H_2) via GATA3 and Foxp3 in a variety of inflammatory conditions (Fig. 2). For the preclinical studies in RA, the secretory factors from adipose MSCs could lead to a reduction in the proinflammatory T helper 17 (T_H_17) cells and its transcription factor RORγt and also increase the regulatory T cells (T_regs_) of RA patients. Besides, the secretory factors from adipose-derived MSCs also show the potential to treat RA by modulating the immune response and reducing inflammation [23]. Cosenza et al. [24] revealed that extracellular vehicles (EVs) generated by MSCs were also crucial paracrine messengers for the regeneration process, which have been proved to embrace anti-inflammatory effects on the local microenvironment.

Several clinical trials have also been conducted in DMARD-refractory patients to evaluate the safety and effectiveness of MSC treatment for RA (Table 2). Clinical research in China (NCT01547091) found that considerable illness remission was followed by lower levels of C-reactive protein (CRP), RF, and anti-cyclic citrullinated peptide (CCP) antibodies after MSC treatment. This study discovered beneficial effects both in 8 months [25] and 3 years [26]. In 2011, Spain launched another randomized multicenter double-blind clinical trial of adipose-derived mesenchymal stem cells (AD-MSCs), which showed similar regulatory effects of MSC therapy for RA treatment (NCT01663116). Although these clinical studies suggest that MSC therapy may hold promise as a potential treatment option for RA, further studies are still needed to determine and optimize the cell source, dosage, and administration route. The safety and efficacy of the treatment must also be confirmed in larger randomized clinical trials. It is important to note that the mechanisms by which MSCs exert their therapeutic effects are not yet fully understood and require further investigation in the future.

Moreover, the present MSCs for clinical trials are administered at relatively high doses. Recent preclinical studies are trying to use biological engineering approaches to deliver MSCs in a controlled manner to effectively treat RA with fewer systemic side effects (Table 3). Several studies also report the potential application of hydrogel for MSC delivery in the treatment of RA. The most common techniques are based on the use of injectable hydrogels to treat RA. MSCs have been reported to be encapsulated in a chondroitin sulfate-based hydrogel and implanted into the knees of rats with RA; the results showed that hydrogel-encapsulated MSCs could reduce inflammation and improved joint function when compared to the control [27]. Another preclinical study by Hu et al. [28] encapsulated the MSC in an alginate/poly-L-lysine/alginate (APA) hydrogel. The results show that hydrogel-encapsulated MSCs could reduce inflammation and cartilage destruction compared to the control group by inhibiting the IL-1 receptor. In conclusion, the preclinical studies and clinical trials of MSC therapy are encouraging and more investigations are still needed to fully establish to achieve the best outcomes for RA treatment.

Macrophages, DCs, and B cells are all involved in the immune response to RA but maintain different roles and functions. In RA, macrophages produce proinflammatory cytokines and chemokines that contribute to synovial inflammation and joint destruction [29]. Recent research has focused on the application of macrophage cell therapy as a potential treatment for RA [30] (Table 4). DCs have been reported to initiate and regulate the immune response in RA. It could activate T cells to alleviate joint inflammation and damage [31,32]. B cells could produce antibodies that target self-antigens, such as citrullinated proteins, which also contribute to joint inflammation and damage for RA [33]. Targeting these cells could help to reduce the cytokine-induced inflammation, further slowing the progression of RA.

**Table 4.** Summary of RA cell therapies.

Cell category	Constituents	Drug	Reference
DCs	Human peripheral blood monocytes	Dexamethasone; vitaminD3; MPLA	[100]
DCs	Human peripheral blood monocytes	Rheumavax	[36]
MSCs	Human bone marrow-derived MSCs	McTNFR2 (Lipofectamine)	[101]
MSCs	Human adipose-derived MSCs	Extract secretory factors from MSC	[23]
Macrophages	Murine macrophage cell line (J774A.1)	IL-10 (nanoparticle)	[30]
Monocytes	Human peripheral blood monocytes	TLR2 (OPN301)	[102]
FLSs	Human synovial biopsies and cell line (MH7A)	TLR4 (TAK-242)	[103]
FLSs	Human synovial biopsies	Chimeric human TNF soluble receptor I (AAV5)	[62]
FLSs	Human, rhesus monkeys, rodents, and rabbits	IFN-β (AAV5)	[63]
B cells	Mouse mononuclear cells	B220 (nanoparticles CRISPR)	[40]
T cells	Human T cells	Citrullinated peptide epitopes (CART)	[39]
T_regs_	Mouse iPSC cell line (MEF-Ng-20D-17)	FoxP3 (retroviruses)	[104]

DCs, dendritic cells; MSCs, mesenchymal stem cells; FLSs, fibroblast-like synoviocytes; IPSC, induced pluripotent stem cells; MPLA, monophosphoryl lipid A; T_regs_, regulatory T cells.

Macrophages can be genetically engineered or manipulated ex vivo to alter their proinflammatory properties and promote an anti-inflammatory phenotype. The depletion of macrophages could prevent synovial tissue infiltration and help the switch from M1 (proinflammatory phenotype) to M2 (anti-inflammatory state). Understanding the complex network of disease-involved cytokines could pave the way for tremendous therapeutic advances by targeting TNF-α, IL-1β, S100A8/A9, GM-CSF, IL-6, IL-10, HMGB, SAA, S100A8/A9, MMP-3, MMP-12, MCP-1/CCL2, CCL3, and CX3CL1, which was produced by macrophages [34]. Recently, Jain et al. [30] described the potential therapeutic effects of tuftsin-modified alginate nanoparticles containing IL-10 for RA. The nanoparticles were able to target and repolarize macrophages in the damaged joints. The yielding of macrophages might decrease the cytokine expression (TNF-, IL-1, and IL-6) to reduce RA inflammation (Table 4).

**Fig. 3.**
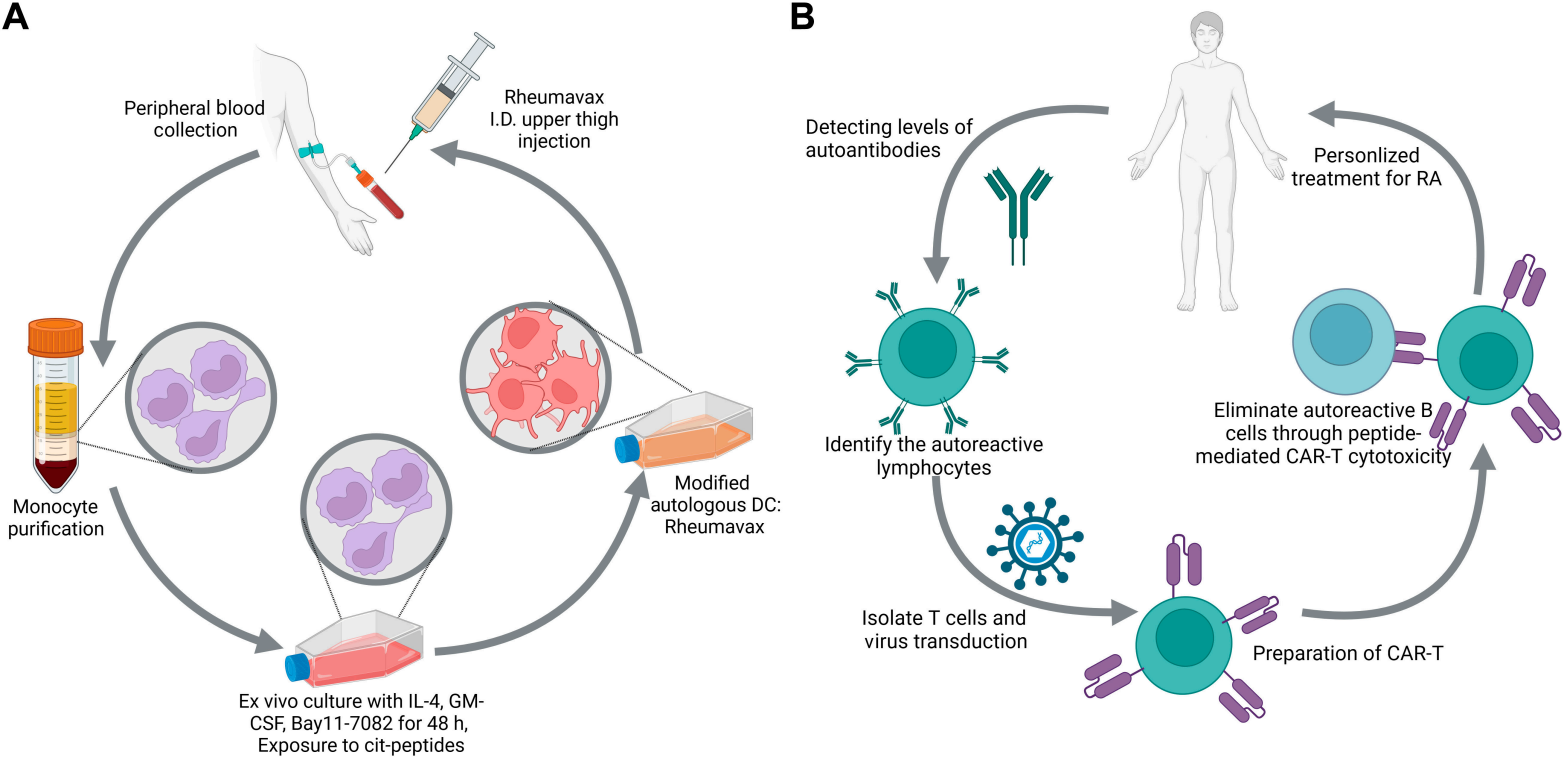
(A) Schematic illustration of immunomodulatory therapy of DCs in patients with RA [36]. (B) Schematic illustration of CAR-T-mediated autoreactive B cell elimination therapy for RA [39].

DCs, as central in inducing immunity and mediating immune tolerance during antigen-presenting process, were shown to be implicated in the progression of RA. It has been found as a critical cell subgroup of treatment resistance in a large-scale immunology study of RA. DC has been studied to be pathophysiologically related to RA therapy response by expressing TGF-β, IL-1, IL-6, IL-12, and IL-23, which are essential for proliferation and further maturation of T_H_1 and T_H_17 cells [35]. Benham et al. [36] described a new immunotherapy approach based on DC for RA patients with specific genetic markers (ACPA or anti-CCP). This suggests another safe and effective therapy for RA patients with this specific genetic marker (Fig. 3A). Rao et al. [37] encapsulated triptolide (TP) with DC-derived exosomes (DEX) to generate a DC-targeted delivery system that could induce immunosuppression of DC and reduce TP toxicity inside RA tissue. The in vivo results showed that encapsulated TP with DEX could alleviate local inflammation and injury in animal models by lowering T cell (CD4^+^) numbers and raising T_regs_. Emerging therapies for RA also exploit the tolerogenic potentials of DCs. The tolerogenic DCs can be generated from myeloid precursors to produce immunosuppressive cytokines and promote crosstolerance with the low production of IL-12 and high production of immunosuppressive mediators, such as TGF-β, IL-10, and indoleamine 2,3-dioxygenase (IDO) [38].

For T cell therapy, Zhang et al. [39] developed chimeric antigen receptor (CAR)-T cells combined with fluorescein isothiocyanate (FITC)–labeled antigenic peptide epitopes. The findings demonstrated that CAR-T cells may be instructed to precisely destroy hybridoma cells and autoreactive B cell subsets by recognizing relevant FITC-labeled citrullinated peptide epitopes from RA patients (Fig. 3B). For B cell therapy, Li et al. [40] developed a library of nanoparticles with varying polyethylene glycol (PEG) concentrations and zeta potentials and then screened for the best nanoparticle for in vivo B cell targeting. Then, they used the optimized nanoparticle loaded with the CRISPR-Cas9 system, which was found to be effective by reducing the B220 genes in B cells in vitro and in vivo. Furthermore, NPCas9/gBAFFR therapy has been reported to reduce the number of B cells and has a therapeutic impact in the treatment of RA.

In conclusion, cell therapy has shown its promising future for the treatment of RA. While preclinical studies have shown promising results, clinical trials evaluating the efficacy of this treatment are needed to establish its effectiveness in patients with RA. Future research still needs to focus on optimizing the delivery system and dosing of cell therapy to achieve the best outcomes for patients with RA.

## Immunoengineering strategies for RA

**Fig. 4.**
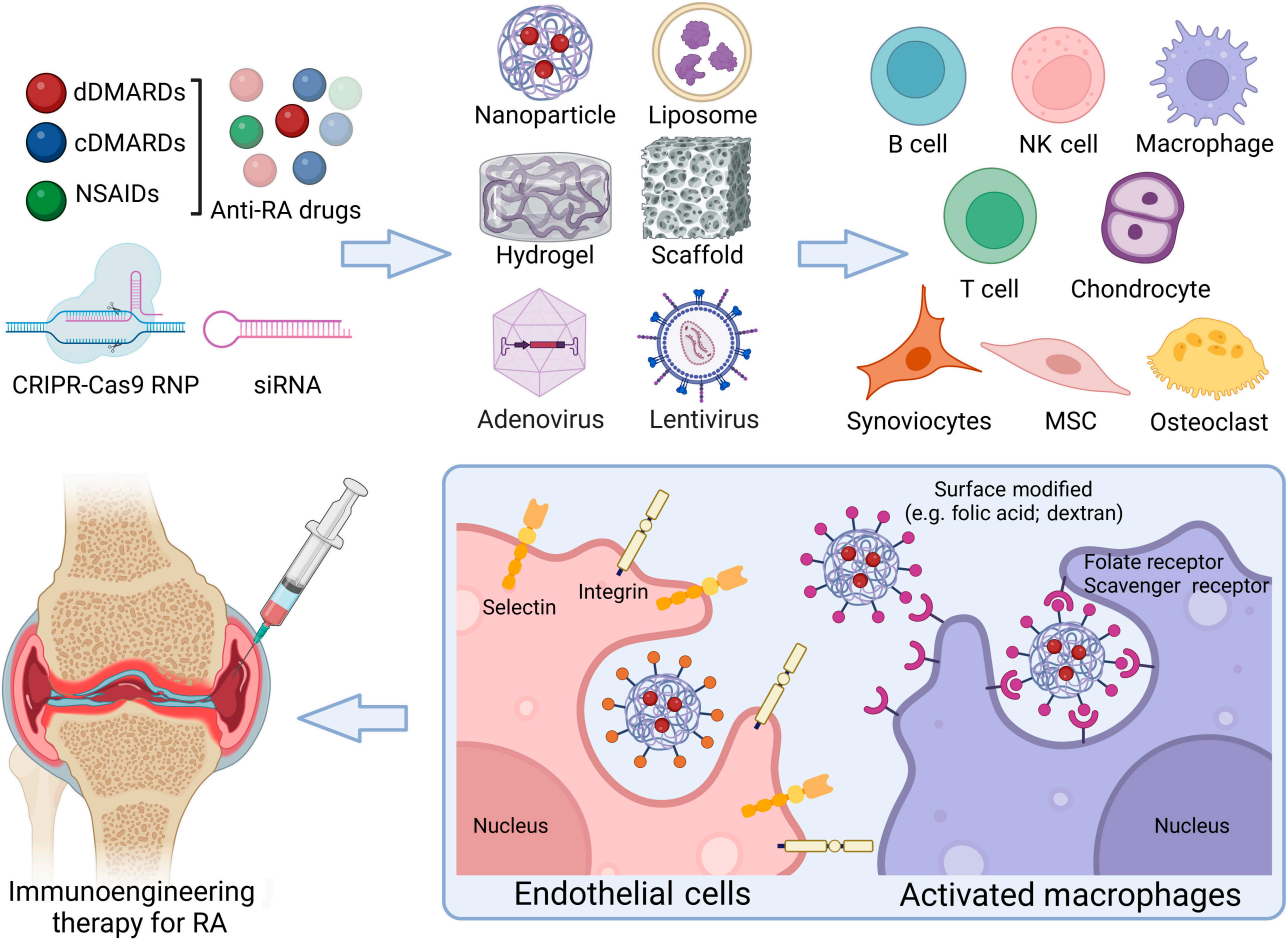
Apart from direct cell therapy, current immunomodulatory strategies could also modify the different kinds of chemicals (e.g., folic acid and dextran) on the surface of the carriers (e.g., nanoparticles, liposome, hydrogel, scaffold, adenovirus, and lentivirus) to improve the affinity to specific targeted cell surface and suppress immune activity in vivo, which might bring a new method for the immunoengineering treatment of RA.

Considering that traditional cDMARDs (e.g., biomacromolecule drugs and cells) are limited by delivery barriers, immunogenicity, and targeting efficiency, it is necessary to use immunoengineering methods to increase delivery efficiency and reduce toxicity. Immunoengineering involves the design and development of immune-active nanotechnology and biomaterials scaffolding through a bottom-up approach using mechanism-based immunology methods, offering a wide range of therapeutic potential for RA treatment. It is based on targeting key pathways in immune cells through immunoengineering methods to harness cellular responses for immunotherapy (Fig. 4) [41].

### Nanotechnology for RA treatment

**Fig. 5.**
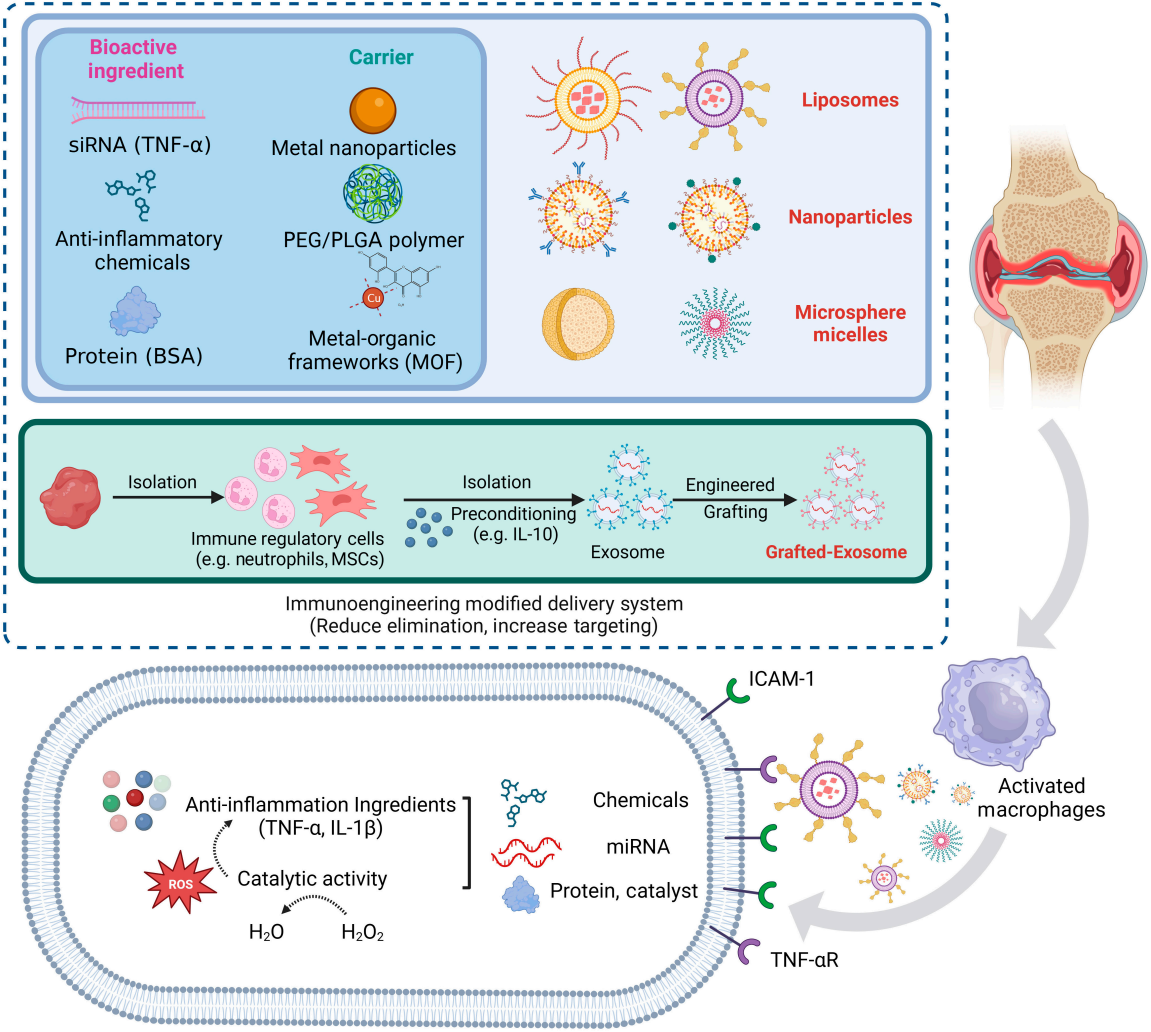
The schematic of immunoengineering modified with nanotechnology targeting the active M1 macrophages for RA to play the intracellular anti-inflammatory effect.

Nanotechnology targeting specific immune cells or tissues is a promising area of immunoengineering therapy. The most commonly used small molecule drugs for treating RA is MTX, which is a folate antagonist that inhibits the proliferation of immune cells [42]. However, MTX has poor bioavailability and can cause side effects such as hepatotoxicity and myelosuppression [43]. Nanotechnology can improve the pharmacokinetics of MTX with the increased enhanced permeability and retention (EPR) effect and reduce its toxicity by encapsulating it in liposomes or polymeric nanoparticles [44]. Nanotechnology could be used to functionalize the biomaterials with targeting moieties, such as antibodies, peptides, or small molecules, to enhance the accumulation in the desired tissue, reduce off-target effects, and protect from degradation, which can improve the efficacy of immunotherapy [45]. So far, several new nanomedicine delivery systems have been investigated, including liposomes, micelles, and nanoparticles, to carry a variety of cargo, such as small-molecule drugs, biologics, and nucleic acids, for immunotherapy of RA to improve the targeting efficiency, decrease dose, and reduce the adverse effects of conventional drugs (Fig. 5) [46].

#### Liposomes and exosomes

Liposomes are small spherical vesicles composed of a phospholipid bilayer that is suitable for the encapsulation of varying solubility profiles (hydrophobic, amphipathic, and hydrophilic molecules), and recent studies showed that liposomes could be used to suppress arthritis with improved accumulation of the drugs and the reduced inflammatory cytokine expression [47,48]. Ren et al. [49] suggested that tailoring the size, surface charges, and PEG length of liposome could significantly enhance the targeting efficiency for dexamethasone therapy. The results show that liposomes could improve the retention of dexamethasone inside the inflamed joints, which was taken by fibroblasts and macrophages. Apart from MTX, the liposome could also be used to deliver the catalase for RA therapy; Chen et al. [50] reported that liposomes made of 1-palmitoyl-2-oleoylphosphatidylcholine (POPC), FOL-S100, and cholesterol could target the activated macrophages and decomposed the hydrogen peroxide into oxygen and water, which eventually increased the therapeutic efficacy of MTX. Glucocorticoids and tofacitinib have also been reported as typical drugs delivered through the liposome [51,52].

Exosomes have exhibited a high propensity to give anti-inflammatory and tissue-repairing effect similar to its parental cells (e.g., MSCs and macrophages). It also has been reported to regulate RA symptoms by secreting miRNAs, protein, and catalyst to reduce infiltration of immune cells into the joint of RA patient [24,53]. Zhang et al. [54] reported a uPB-Exo modified from neutrophil-derived exosomes modified with ultrasmall Prussian blue nanoparticles via click chemistry, which could activate the FLSs to reduce the inflammatory events via the T_H_17/T_reg_ signal to further ameliorate joint damage. Tang [55] also reported a novel anti-inflammatory exosome (Al-Exo) made from exosomes and IL-10 to play its anti-inflammatory effect by promoting macrophage polarization to M2 phenotypes to relieve inflammation symptoms and accelerate tissue repair against RA.

#### Nanoparticles

Nanoparticles are small particles in the range of 1 to 100 nm, which can be made from metals, polymers, lipids, and proteins that make them useful in a wide range of applications for RA treatment. Lyu et al. [56] incorporated MTX into mannose-modified MTX-M-NP nanoparticles to target the medication to neutrophils. The findings demonstrated that MTX-M-NPs might reduce inflammatory cytokines and bone degradation in RA [57]. B cell is also an important type of immunity cell in the immune response and development of RA. Wu et al. [58] described a potential treatment approach for RA using nanoparticles to deliver small interfering RNA (siRNA) (siBAFF) to target the BAFF receptor (BAFF-R) in B cells. It could decrease the B cells inside the joint to inhibit inflammation processes. The approach may also reduce blood anti-collagen immunoglobulin G (IgG) levels while increasing collagen type II and osteocalcin expression in dissected joint tissues. Jhun et al. [59] described hybrid nanoparticles composed of liposomes and gold encoded with CoQ10 (LGNP-CoQ10) to target the signal transducer and activator of transcription 3 (STAT3)/T_H_17 pathway, which is involved in the inflammatory response. These findings suggesting that LGNP-CoQ10 is efficient for the treatment of RA.

In addition to traditional nanoparticles, other active nanoparticles represented by stable metal organic framework viruses have also been investigated for immunoengineering therapy of RA [60]. Li et al. [61] created chondroitin sulfate-based prodrug nanoparticles that transported the photosensitizer chlorin e6 as well as retinoic acid to minimize photodynamic therapy (PDT)-mediated immunosuppression by disrupting the Golgi apparatus and preventing the synthesis of immunosuppressive cytokines. Apart from nanoparticles, Adriaansen et al. [62] modified adeno-associated AAV5, which encodes a chimeric TNF-α linked to immunoglobulin heavy chain Fc portion (rAAV5.NFkB-TNFRI-Ig). The results show that the production of the TNF-blocking agent in response to disease activity could reduce the potential for side effects. Similarly, preclinical study on the use of AAV5 vector to deliver human interferon-β (IFN-β) to treat RA also showed a reduction in inflammation and joint damage, which suggests that hIFN-β produced by RA has the potential to be an effective and targeted therapy for RA with minimal off-target effects [63].

#### Microspheres and micelles

Microspheres and micelles have been widely used in drug delivery, where they can be engineered to encapsulate drugs or other bioactive molecules to be delivered into RA tissues. Microspheres are typically solid or hollow spherical particles, while micelles are amphiphilic molecules, such as lipids or surfactants, which could self-assemble in water as emulsifiers to solubilize poorly soluble drugs or molecules. Erdemli et al. [64] reported that MPEG-PCL-MPEG microspheres were able to preserve encapsulated etanercept for 90 days while also significantly lowering proinflammatory cytokines (TNF-α, IFN-c, IL-6, and IL-17) and MMP levels (MMP-3 and MMP-13) (Table 5). Bassin et al. [65] developed a microsphere (TRI-MP) made from polylactic-co-glycolic acid (PLGA) and mPEG to deliver TGF-β, rapamycin, and IL-2 (TRI). The results showed that intra-articular injection of TRI-MP could effectively reduce arthritis incidence, the severity of arthritis scores, and the erosion of bone, suggesting that TRI microparticles may have potential as a therapeutic option for RA. Wang et al. [66] developed a micelle system made from PCL-PEG to improve the efficacy of low-dose glucocorticoid therapy. The micelles have been found to encapsulate dexamethasone and target inflamed joints. The results showed that the micelles could effectively reduce inflammatory cytokine expression in RA. Xu et al. [67] described a micelle made from octadecanoic acid-grafted dextran and sialic acid. The micelles could encapsulate MTX to significantly inhibit the inflammatory response and diminish the adverse effects of MTX. Moreover, the study suggests that micelles could also enhance bone regeneration and repair by promoting osteoblast differentiation and mineralization.

**Table 5.** Current nanotechnology therapies for RA.

Biomaterial category	Constituents	Drug	Therapeutic action	Model	Route of administration	Advantage
Liposome	•Egg yolk lecithin	DEX	CIA	ICR mouse	iv	•Anti-arthritic efficacy [49]
	•Cholesterol					
	•PEG					
Liposome	•POPC	MTX; catalase	CIA	C57BL/6 mice	iv	•Prolonged blood circulation time.
	•FOL-S100					•Enhanced accumulation of MTX in inflamed joints.
	•Cholesterol					•The minimal toxicity [50].
Liposome	•Liposomal glutathione	Glutathione	Pristane-induced arthritis	Wister Albino rats	id	•Reduced rheumatoid factor, malondialdehyde, and C-reactive protein levels [48].
Liposome	•Egg phosphatidylcholine	Ag; curcumin, quercetin, Bay11-7082	-	C57BL/6 mice	-	•Adapted to deliver Ags and inhibitors for Ag-specific suppression [105].
Liposome	•SA-CH	DEX; palmitate	AIA	Wistar rats	iv	•Reinforced accumulation of drug in peripheral blood neutrophils (PBNs) [106].
	•DP-SAL				
Liposome	•DSPC	MTX; p-coumaric acid	AIA	Wistar rats	iv	•Inhibits osteoclast formation and bone resorption [107].
	•Cholesterol					
	•Mannose					
Liposomes	•DSPC	MTX; prednisolone	-	White Albino rats	iv	•Superior targeting efficiency [47].
Liposomes	•Cholesterol	DEX	AIA	Sprague-Dawley rats	iv	•Effectively suppressed the joint swelling.
	•Stearylamine					•Significantly down-regulated serum proinflammatory cytokines [108].
Liposomes	•Cholesterol	Tofacitinib citrate	-	Wistar rats	iv	•Improved accumulation in RA tissues; reduced the inflammatory cytokine expression and lipid peroxidation [52].
	•SPC					
Liposomes	•Phosphatidylcholine HSPC	Glucocorticoid; betamethasone; methylprednisolone	-	Lewis rats	iv	•Suppressed the secretion of proinflammatory cytokines.
	•Cholesterol					•Not change the levels of TGF-β [51].
	•PEG					
	•DSPE					
Liposomes	•DC8,9PC	DEX	AIA	SD rats	iv	•Suppressing the level of proinflammatory cytokines (TNF-α, IL-1β) in joint tissues.
	•DSPE-PEG					•Reducing the swelling of inflamed joints and alleviating the progression of RA [109].
Microparticle	•PEGDA	TSG-6	MMT injury	Sprague-Dawley rats	ia	•Reduced cartilage damage [110].
	•Dithiothreitol					
	•BSA					
Microparticle	•PLGA	TGF-β; rapamycin; IL-2	CIA	DBA/1J	sc	•Effectively reduced arthritis incidence, the severity of arthritis scores, and bone erosion [65].
	•mPEG					
Microparticle	•mPEG	Etanercept	-	Fibroblast-like synoviocytes		•Significant decrease in proinflammatory cytokines (TNF-α, IFN-c, IL-6, IL-17) and MMP levels (MMP-3, MMP-13) [64].
	•PCL					
Mielle	•PCL	DEX	Adjuvant-induced arthritis	Wistar rats	iv	•Potently reduced joint swelling, bone erosion, and inflammatory cytokine expression in both joint tissue and serum [66].
	•PEG					
Mielle	•RGD	MTX; nimesulide	AIA	SD rats	iv	•Enhance the effect on rheumatoid arthritis therapeutic [111].
	•PEG					
	•PLA					
Mielle	•Octadecanoic acid-grafted dextran	MTX	AIA	Wistar rats	iv	•Significantly inhibited the inflammatory response, but also diminished the adverse effects of MTX [67].
	•Sialic acid					
Nanoparticles	•Human serum albumin	MTX	CIA	Sprague−Dawley rats	iv	•Decreased inflammatory cytokines, joint swelling, and bone erosion [57].
Nanoparticles	• α-Cyclodextrin	MTX	CIA	DBA/1 J mice		•Significantly accumulated at inflamed joints.
	•CDI-activated HPAP					•Alleviated the joint swelling and cartilage destruction.
	•Folic acid					•Inhibited the expression of iRhom2, TNF-α, and BAFF [112].
Nanoparticles	•PEG-PLGA	BAFF-R; siRNA	CIA	DBA1J mice	iv	•Decreasing the arthritis score, mean ankle diameter, the levels of anti-collagen IgG in serum.
	•Cationic lipid DOTAP					•Increasing the expression of collagen type II and osteocalcin in dissected joint tissues.
						•Decreased the percentage and number of B cells.
						•Inhibited the production of proinflammatory cytokines [58].
Nanoparticles	•DSPC	Coq10	CIA	DBA/1J mice	-	•Decreased proinflammatory cytokines (IL-17); T_H_17 cell and phosphorylated STAT3-expressed cell populations [59].
	•Ascorbic acid					
	•Gold precursor aqueous solution					
Nanoparticles	•mPEG-PLGA	Benzoylaconitine	Egg white-induced mice paw edema	Kunming mice	iv	•Substantially inhibiting secretion of proinflammatory cytokines [113].
Nanoparticles	•PLGA	Resveratrol	CIA	C57BL/6 mice	In situ injected	•Effectively accumulate in the lesion area.
	•Dextran sulfate					•Enhanced the transformation of the M2 type macrophages from M1 [114].
	•Ruthenium					
Nanoparticles	•Glycol chitosan	MTX	AIA	Wistar mice	iv	•High accumulation in inflamed joints [115].
	•Steric acid					
Nanoparticles	•FA	AgNO_3_	AIA	DBA/1J mice	iv	•Passively accumulate into inflamed joints
	•LA-PEG					•Anti-inflammatory activity [116].

AIA, Freund’s adjuvant-induced arthritis; BAFF-R, B-cell activating factor receptor; CIA, collagen-induced arthritis; Coq10, coenzyme Q10; DEX, dexamethasone; DP-SAL, liposomal dexamethasone palmitate; DSPC, 1,2-distearoyl-sn-glycero-3-phosphocholine; ia, intra-articular injection; id, intradermal injection; iv, intravenous injection; MMT, medial meniscal transection; MTX, methotrexate; mPEG, methoxy poly(ethylene glycol); PCL, polycaprolactone; PEG, polyethylene glycol; PLA, polylactic acid; PLGA, polylactic-co-glycolic acid; SA-CH, sialic acid–cholesterol conjugate; sc, subcutaneous injection; TSG-6, TNF-α-stimulated gene-6.

Together, due to the low therapeutic concentration in joints and systemic side effects of orally administered RA therapeutics, DMARDs have been encapsulated through nanotechnologies into various carriers (liposomes and exosomes, nanoparticles, microspheres, and micelles). The nanotechnologies outweigh other conventional methods in their controlled drug release and reduced systemic toxicity, which could significantly enhance the effectiveness of current DMARDs for RA. However, considering that RA’s mechanism is complicated, future work would focus on making more than one target to block several pathways simultaneously during the RA pathogenesis pathways. More kinds of nanomedicines with low dose, minimal adverse effects, and improved therapeutic efficiency would be innovated for RA.

### Hydrogel biomaterials for RA

Hydrogels are three-dimensional (3D) networks of crosslinked hydrophilic polymers that have the ability to absorb huge volumes of water while retaining structural integrity [68]. Hydrogels can be synthesized from natural and synthetic polymers, including collagen, hyaluronic acid, and PEG. They have several properties that make them appealing for use as scaffold materials in tissue engineering, including high water content, biocompatibility, and the ability to mimic the extracellular matrix of native tissue [69]. Also, hydrogels exhibit a variety of properties, including mechanical strength, degradation rate, and biocompatibility to mimic the microenvironment of tissues [70], providing support for cell growth, proliferation, and differentiation [68,71]. These characteristics make them an attractive option for immunoengineering applications in RA (Table 6) [72].

**Table 6.** Current hydrogel biomaterial therapies for RA.

Biomaterial category	Constituents	Drug	Therapeutic action	Model	Route of administration	Advantage
Hydrogel	Tetrazine (TET)Transcyclooctene (TCO)Hyaluronic acid (HA)	MTX	Collagen-induced arthritis (CIA)+AIA	RA rat	Intra-articular injection	Most significant RA reversal; increased cartilage thickness, extensive generation of chondrocytes and glycosaminoglycan deposits, extensive new bone formation; suppression of TNF-α and IL-6 [103]
Hydrogel nanoparticles	Disulfide-crosslinked polyethyleneimine (PEI-SS);F127;F68	IndomethacinMethotrexateMMP-9 siRNA	Collagen-induced arthritis	DBA/1J mice	Intra-articular injection	Relieved joint swelling and significantly reduced the expression of TNF-α, IL-6, and MMP-9 in the joint fluid [75]
Hydrogel	TG-18	Triamcinolone acetonide (TA)	K/BxN serum-induced arthritis	C57BL/6J mice	Intra-articular injection	Reduces arthritis activity [76]
Hydrogel nanoparticles	Poloxamer 407 (P407); PCL; PVA; PEG	Tacrolimus (FK-506)	Adjuvant-induced arthritis (AIA)	Sprague–Dawley rats	Subcutaneously injected near the swollen ankles	Longer retention time; the significant inhibition of edema [77]
Hydrogel	Pluronic F127 HA	Infliximab	Ovalbumin (OVA) + AIA	New Zealand white rabbits	Intra-articular Injection	Alleviate the expression of inflammatory cytokines, such as TNF-α, IL-1β, IL-6, and IL-17, relieve pain, inhibit cartilage destruction [78]
Hydrogel nanoparticles	Disulfide-crosslinked polyethyleneimine (PEI-SS);F127;F68	IndomethacinMethotrexate	Collagen-induced arthritis	Wistar rats	Intra-articular injected	Reduced joint swelling, bone, erosion, and expression of inflammatory cytokines [117]
Hydrogel	3D printed porous metal scaffolds;Hyaluronic acid-hydrazide	Infliximab, ADSC	Ovalbumin (OVA) + AIA	New Zealand white rabbits	Scaffold was implanted into the predrilled defect	Down-regulate inflammatory cytokines, rebuild damaged cartilage, as well as improve subchondral bone repair [79].
Hydrogel	TYRAMINE-modified gellan gum;Silk fibroin	Betamethasone	Lipopolysaccharide (LPS)	THP-1 cell line	In vitro model	Increase therapeutic efficiency [80]

Hydrogels can encapsulate NSAIDs and DMARDs and deliver them locally to joints, reducing systemic toxicity and increasing drug efficacy to target sites in addition to being loaded with growth factors or other bioactive molecules that can promote cell proliferation and differentiation while modulating the immune response [73]. Seo et al. [74] reported the efficacy of a click-crosslinked hyaluronic acid (HA) depot for injection in prolonging RA treatment actions in joints. The results found that HA could form a stable and elastic hydrogel after injection, and sustained release of the loaded MTX could persist. The constructed MTX-Cx-HA hydrogel could improve articular index scores, increase cartilage thickness, generate extensive chondrocyte and glycosaminoglycan (GAG) deposits, and suppress the expression of TNF-α and IL-6. Yin et al. [75] developed a PEI-SS-IND-MTX-MMP-9 siRNA nanoparticle loaded with indomethacin (IND), MTX, and siRNA to target MMP-9 to sustain drug release. The nanoparticles were then encapsulated by poloxamer hydrogel (F127 and F68). The results show that the hydrogel system could relieve the expression of TNF-α, IL-6, and MMP-9 in the joint fluid, which might be a promising approach for the synergistic treatment of RA. Joshi et al. [76] developed a drug delivery hydrogel system made of TG-18, which could self-assemble to form arthritis flare-responsive hydrogels. They reported that the platform could deliver triamcinolone acetonide (TA) and exhibit sustainable drug release characteristics to reduce arthritis activity. Therefore, the injectable hydrogel may be a promising approach to prolong therapeutic activity in treating RA.

Thermosensitive hydrogel has also shown its good prospective in sustained-release properties for RA. Wu et al. [77] proposed a drug delivery hydrogel system for the treatment of RA: a cross-linking thermosensitive hydrogel (Soluplus) composed of poloxamer 407, PCL, polyvinyl alcohol (PVA), PEG, and tacrolimus (FK-506). The longer retention time and the significant inhibition of edema for this system suggest that this system has the potential to provide improved treatment outcomes with fewer side effects compared to existing treatments. Chen et al. [78] investigated the thermosensitive hydrogel composed of pluronic F127 and HA with polyglycolic acid (PGA) by incorporating infliximab (IFX). The results demonstrated that IFX-loaded hydrogel could decrease the inflammatory cytokines in synovial fluid and cartilage, as well as ease pain and limit cartilage deterioration in RA. These results suggest that this treatment has the potential for use in patients with RA. Similarly, Zhao et al. [79] reported research exploring a temperature-sensitive hydrogel (D-NGel), which could simultaneously deliver IND and MTX in combination to reduce inflammatory cytokines. Oliveira et al. [80] produced a hydrogel made of tyramine-modified gellan gum with silk fibroin (Ty–GG/SF). The results found that the hydrogel effectively sustained the release of betamethasone.

**Fig. 6.**
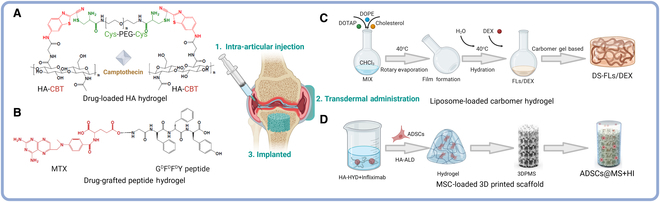
Schematic illustration for the synthesis of (A) HA hydrogel [81], (B) MTX-GDFDFDY hydrogels [82], and (C) DS-FLs/DEX hydrogels [83], which could exert anti-inflammatory effect by sustaining the release of camptothecin, MTX, and DEX. (D) Schematic illustration showing the fabrication process of self-healing infliximab-based hydrogels combined with 3D printed porous metal scaffolds (3DPMS) to deliver ADSCs in aiding RA management.

DMARDs could also be designed to graft into the hydrogel’s backbones through click chemistry reaction. Recently, Gao et al. [81] introduced an intra-articular drug delivery system with camptothecin nanocrystals made by naturally occurring click chemistry (Fig. 6A). The results show that the synthetic HA hydrogel could release free camptothecin in a sustained manner and alleviate arthritis. Ma et al. [82] developed a supramolecular self-assembling hydrogel GDFDFDY with conjugated MTX into MTX-GDFDFDY hydrogels to significantly alleviate RA syndromes of joint swelling to protect cartilage and show its potential therapy for RA (Fig. 6B). Zhao et al. [83] developed a flexible liposome hydrogel (DS-FLs/DEX) hydrogel made from transdermal formation dextran sulfate (DS) and DEX. The results suggest that DS-FLs/DEX demonstrated sustained drug release and efficiently penetrated and accumulated in inflamed joints to diminish inflamed joints and the detrimental effect of RA on bone, proving to be a good drug delivery vehicle against RA (Fig. 6C).

The synthesis hydrogel could also integrate with the 3D printing technology and microfluidic microsphere preparation technology to construct 3D structure with designed shape and ingredient. Zhao et al. [84] created a self-healing IFX-based hydrogels combined with 3DPM to protect the implanted cells from hypoxia-induced apoptosis by delivery with adipose-derived stem cells (ADSCs) (Fig. 6D). It could also integrate with 3D printed microporous titanium alloy scaffold to suppress the local inflammatory cytokines and improve osseointegration. It offers a solution to the long-standing problem of stem cell transplantation to promote prosthetic interface osseointegration in RA. Apart from the 3D printed scaffold, the hydrogel itself could also be applied into microsphere delivery system [72]. Li et al. [85] developed an injectable sustained-release dACC/dAPC-DSPE-PEG-FA microspheres loaded with TNF-α siRNA/artemisinin. The biodegradable HA microsphere has been loaded with nano-lipoplex made from TNF-α siRNA and artemisinin. The result show that the biodegradable HA hydrogel microsphere could sustain activity of gene therapy and artemisinin efficacy for RA long-term treatment.

Together, hydrogels have emerged as a promising strategy for tissue engineering in RA and can be tailored to exhibit a variety of properties and have been utilized for drug delivery, cell delivery, and scaffold materials in preclinical studies. Hydrogels have great potential for the development of effective RA treatments with minimal side effects. Although there are promising applications for hydrogels in RA immunoengineering, there are several limitations that need to be addressed. One major challenge is the lack of long-term stability of hydrogels in vivo, as they can undergo rapid degradation or clearance by the immune system. In addition, the complexity of the synovial joint microenvironment, including the presence of various immune cells and cytokines, also presents a challenge for the design of hydrogels that can effectively modulate the immune response [86].

## Conclusion and Perspectives

RA is a complex autoimmune disease. Current therapies (e.g., NSAIDs or DMARDs) that have been used to treat RA aim to suppress or modulate the activity of the immune system. However, present clinical methods are severely hampered by a number of flaws, including significant systemic adverse effects, nonspecific targeting, frequent administration routines, and high costs. Furthermore, the lack of effectiveness and selectivity for targeted receptors in RA also hampered the pharmacological development and clinical application.

To address these issues, immunoengineering strategies employ biomaterial hydrogels, microparticles, and nanoparticles that could generate an immunosuppressive delivery vehicle to target and modulate the immune response in RA. However, there are still significant gaps in understanding the transport and delivery mechanism for the nanocarriers to be effectively delivered into the inflamed joints. More studies are required to define the particular features, such as ideal size and surface characteristics, of these nanocarriers to facilitate more specific and targeted delivery and reduced off-target effects.

**Fig. 7.**
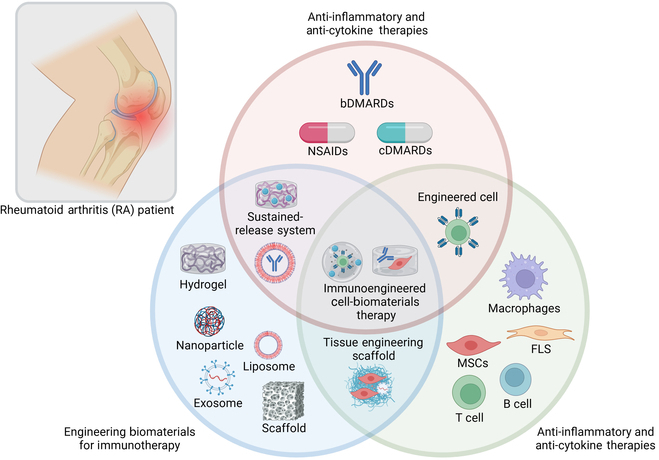
With the inclusion of anti-inflammatory therapy, the engineering biomaterial therapy, and the anti-cytokine cell therapy, the more specific RA immunoengineering therapy could be invented to offer potential possibilities to RA patient.

Continued improvement upon biomaterials and cell-instructed immunomodulatory therapies would depend upon increasing understanding of the regulatory mechanism of cytokine factors and related pathways, the cell–matrix and cell–material interaction, and the innovation of genetic engineering vehicles. The solution for the above problems might largely depend on the inspiration from cancer immunotherapy, which might be readily applied to musculoskeletal problems to design novel solutions through genetically tool synthetic immunology and mechanobiology. Application for the current DMARDs along with the genetic engineered cells and the de novo designed scaffold or cargos would not only bring hope for the improvement of our current immunomodulatory strategies but also broaden our understanding of the immune microenvironment in musculoskeletal disease pathogenesis. We could foresee more kinds of immunoengineering therapy with decreased dosing, and dosing frequency will be available for clinical trials. Furthermore, large-scale profiling of immunomodulatory factors, including cytokine profiles, signaling pathways, and cell–biomaterial interactions for RA, would help in understanding the molecular mechanisms to guide the novel design of more efficient therapies (Fig. 7).
